# Medical Intelligence

**Published:** 1848-01-01

**Authors:** 


### Medical Intelligence.

### CHEMICAL PATHOLOGY IN RELATION TO THE DISEASES OF THE BRAIN AND NERVOUS SYSTEM.

Dr. Bence Jones has been publishing in "The Lancet" a series of papers on this highly important and deeply interesting subject, which are worthy the attention of all psychologists. Our contemporary says:—

"Dr. Golding Bird had observed cases in which excessive mental exertion has been attended by an increased elimination of the triple phosphates by the kidneys; but Dr. Bence Jones has, we may say, proved, that in acute idiopathic inflammation of the brain, and in traumatic cerebritis, the amount of phosphates in the urine is excessive —the phosphoric compounds in the urine of course repesenting the chemical changes going on in the phosphorized cerebral matter, in consequence of inflammatory disease. This deduction from an extensive class of facts offers a very beautiful and striking instance of the intimate relation which exists between pathology and organic chemistry."

The following are the conclusions to which Dr. Jones has arrived on this important subject:—

"Paper I.—The variations of the earthy phosphates are so dependent on the earthy matter present, that no deduction from them of the nature or state of the disease is possible.

"Paper II.—Neither the earthy phosphates nor the alkaline phosphates are increased in spinal diseases.

"Paper III.—In fevers and acute inflammations of fibrous, muscular, or cartilaginous tissues, the amount of earthy and alkaline phosphates together is not increased.

"Paper IV.—In chronic diseases, in which the nervous tissue is not affected, no deduction can be made.

"Paper V.—Chronic cases of general paralysis, mania, and melancholia give no marked results.

"Paper VI.—In chronic diseases of the brain, and in chronic or even acute disease of the membranes, there is no increase of earthy and alkaline phosphates together.

"Paper VII.—In fractures of the bones of the skull, when any inflammation of the brain appears, there is an increase of the phosphates. When there are no head symptoms, no increase of phosphates is observed even when other acute inflammations supervene.

"Paper VIII.—In acute inflammation of the brain there is an excessive amount of phosphates excreted; when the acute inflammation becomes chronic, no excess of phosphates is observed.

"Paper IX.—In some functional diseases of the brain an excessive amount of phosphates is observable. This ceases with the delirium. Delirium tremens shows a most striking deficiency in the amount of phosphates excreted when no food is taken. When food can be taken the diminution is not apparent.

"Hence the general deductions are—

"1st. That acute affections of the nervous substance, organic and functional, are the only diseases in which an excess of earthy and alkaline phosphates appear in the urine.

"2nd. That the amount of phosphates is sometimes excessively diminished in delirium tremens.

"3rd. That no chronic diseases show any marked excess in the total quantity of phosphatic salts excreted—that is, so far as this mode of analysis is conclusive. Mollities ossium in its most active form is an exception to this deduction.

"It seems, then, probable that the action of oxygen on the nervous substance is lessened by alcohol, and that by acute inflammation or excessive excitement of the brain it is pre-eminently increased."

---

### MEDICAL SOCIETY OF LONDON.

MONDAY, NOV. 28, 1847.—MR. DENDY, PRESIDENT.

(*From the Lancet.*)

### CEREBRAL DISEASE IN CONNEXION WITH DISEASE OF THE EAR.

MR. PILCHER exhibited a portion of the brain of a child who had been the subject of ear disease. The child sunk immediately, from acute abscess in the left hemisphere of the brain consequent upon the ear disease. On examination after death, an abscess was discovered in the lateral ventricle, communicating with the other collection of matter. This latter abscess bore the appearance of being of long standing, and was surrounded by a thick, dark, lining membrane. The question to be decided was, whether this latter abscess was an old or a recent formation; if old, it most probably had existed for years. To show the way in which disease of the brain might occasionally, for a time, be masked, or the symptoms suspended, and the disease afterwards prove suddenly fatal, he related the case of a little girl who had suffered from ear disease, attended by much purulent discharge. Convulsions attended the disease, but these became much less frequent, and she recovered almost her usual health. She was so much better, indeed, that the practitioner in attendance was puzzled. The patient one day went to sleep and never awoke. An abscess was found occupying one of the lobes of the cerebellum, capable of containing four ounces of pus, and surrounded by a distinct cyst.

Mr. HIRD remarked that the experiments of Mr. Mayo had seemed to establish that when pressure was exerted downwards, as in the last case related, paralysis was almost always the result; but when the pressure was exerted laterally, no symptoms might present themselves. He (Mr. Hird) had seen cases in which disease of the cerebrum and upper portion of the brain was present without any, or with but few symptoms; when the disease was situated at the base of the brain, involving the medulla oblongata, symptoms were always present.

Dr. BENNETT related a case of extensive disease involving a greater portion of the medulla oblongata, which was not attended by symptoms of that mischief, until within a few hours of the death of the patient. The child was of healthy appearance. Squinting was the first symptom which developed itself—this was followed by convulsions. After death, a tubercular mass was discovered, involving the greater portion of the medulla oblongata, and as large as a full-grown walnut.

DR. CHOWNE related two cases to show that disease of the brain might exist to a considerable extent without any evidence being exhibited of the seat of the mischief. The first of these cases was that of a young woman who was admitted into Charing Cross Hospital with what might be called general illness. She had acute headach; fever, sometimes remitting, sometimes intermitting, and a peculiarity of expression of the face. She died, and an abscess, affecting one hemisphere of the brain, was discovered. In the second case the patient was a young man, admitted into the hospital "very ill," but complaining chiefly, if not entirely, of pains about the right side of the neck, like rheumatism. The neck was fomented. The countenance was peculiar, and such as he had noticed before in cases of brain disease. He suffered from severe inflammation of the ear, suppuration came on; he lived several weeks, but had no pain in the head. A small abscess was discovered situated at the upper part of the middle lobe of the cerebrum. A yellow, thick, tenacious pus was found in the abscess. He referred to a case, which had been related to the Society some time back, of a merchant who continued to conduct an extensive and anxious business to within two or three days of his death, and on examination it was found that the anterior lobes of each hemisphere of the brain were softened to a considerable extent.

---

### PROPOSED ASYLUM FOR IDIOTS.

A PUBLIC meeting in favour of establishing an asylum for idiots was held October 27th, 1847, at the London Tavern, at two o'clock.

The Right Hon. the LORD MAYOR was called to the chair, and opened the proceedings by a brief address in support of the objects of the meeting. In this great metropolis (observed his lordship) charities of every kind and description abounded for every possible purpose, with the single exception of reclaiming and harbouring those whom Providence had not blessed with the entire use of their faculties. He trusted that meeting would do something to fill up the blank, and foster and cherish the object for which they had assembled, so that it should become one of the great charities for which the city of London was so justly celebrated. There was not a market-place in the kingdom wherein some poor idiot did not wander about exposed to the laughter and jeers of an unreflecting populace. If this meeting should succeed in establishing an asylum where these poor creatures could be brought together, and improved, as far as possible, in their mental condition, they would render no small service to their country and humanity.

Mr. GILPIN read the address of the provisional committee.

Mr. WIRE announced that Sir J. Forbes had sent a donation of 100*l.*, that Lord Dudley Stuart and Sir P. Laurie had promised their assistance and patronage, that the Lord Mayor had given a subscription of 21*l.*, and then proceeded to propose the first resolution. It was as follows:—"That in the judgment of this meeting, it is most desirable an asylum be provided for the care and education of the idiot, and that it be forthwith begun." He vindicated the objects of the meeting, the time at which it was assembled, and argued that it had been proved, by actual experiment on the continent, to be quite possible to restore the idiot to the use of his faculties, and to consciousness of mind and soul. Medical science had demonstrated the fact, that by a judicious course of training, the idiot could be made a useful member of society, and even were that a doubtful matter, it was incumbent on the community to provide an asylum for the wretched creatures who were now exposed to ridicule and mockery in our streets. After some further observations, the learned under-sheriff sat down amid applause.

Dr. LITTLE seconded, and Dr. BROWNE briefly supported it.

The Rev. Dr. REID read the rules, which were adopted.

On the motion of the Rev. W. CHAMPNEYS, who addressed the meeting in an eloquent speech, and cited some interesting examples of the impression which religious truths made on the minds of the most idiotic;

Mr. WILKS, chairman of the Hanwell Lunatic Asylum, in seconding the motion of the reverend gentleman, took occasion to declare that the idiot population were not in such a state of destitution as might have been inferred from the speeches of those who had preceded him; but that, on the contrary, a large proportion of those admitted into the various lunatic asylums were afflicted with mental imbecility. He did not wish to argue that the contemplated asylum would not be highly useful, but merely to show that the idiotic part of the patients in the existing institutions were not completely neglected, but were, on the contrary, treated with the greatest attention and regard for their physical wants.

Mr. GEORGE THOMPSON proposed a list of names for the adoption of the meeting as patrons, vice-presidents, and members of the committee; and contrasted the anxiety and kindness with which the idiot was treated by pagans, like the Chinese or Turks, with the neglect exhibited towards them by ourselves, as an argument in favour of the establishment of the asylum, which he hoped to see multiplied throughout the land.

The Rev. Dr. CARLILE seconded the adoption of the names, which were approved of by the meeting.

After some further routine proceedings, and a vote of thanks to the Lord Mayor, the meeting separated.

(As the subject is one of great importance, we purpose in our next number giving a more detailed account of the speeches on the occasion.—ED.)

---

### SELECTED DESIGN OF THE NEW MIDDLESEX PAUPER LUNATIC ASYLUM.

(From *The Builder* of Dec. 11, 1847.)

THE land on which the asylum is to be erected is situate at Bet's Stile, near Colney Hatch, between Finchley Common and Southgate. It consists of about 119 acres, lying on both sides, but chiefly on the west side of the Great Northern Railway, and has a sharp slope to the south-east. On the north side is a lane, which runs from Colney Hatch to Bet's Stile. The first selected plan (by Mr. Daukes,* premium, 300*l.*), or rather plans, for there are three,—one for a two-story building, one for a three-story building, and a third, partly of one and partly the other,—has the entrance-front next this lane, and presents a long, straight line of building, from east to west, with wings at each extremity, extending from the central line on the two-story plan both north and south, and on the three-story plan to the north only. The offices are on the south side, and will interfere with the appearance of the building, as seen from the railway. The plan appears to have been well considered; the galleries are light and airy, chiefly single-warded, and the arrangements generally good; but the building being spread out so far as it is, the distance to the kitchen from some of the wards is very great. Externally, little has been aimed at: the dwelling-house style has been adopted (rusticated quoins and a dentil cornice), with a plain bell turret in the centre of the main building. In one of the perspective views the building is shown to be roofed in compartments, and has the appearance of consisting of a number of distinct buildings brought together. The estimated cost of the building, if the accommodation be obtained in two stories, is 85,000*l.*; if in three stories, 75,000*l.*

It is right we should say that we have a personal bias in favour of the second design selected (by Mr. G. Godwin and Mr. Harris,† premium, 200*l.*), and leave our readers to form their own judgment. This bias, while it aids, perhaps, in leading us to give unqualified preference to the second design, both as regards arrangement and architectural treatment, forbids us to do more than describe it. The plan is the H shape on a large scale, two stories in height, with three short lateral projections towards the east, and three towards the west, giving the means for very perfect classification and separation. The entrance is in the centre of the south front, to which importance is given by the chapel, which projects forward, and a clock tower rising from the main building behind.

In this design the females occupy the western division, and the males the eastern division; these are connected with the chapel and central offices by wards for aged and quiet patients on the female side, and by a passage at the back of various domestic offices and workshops on the male side, as well as by verandahs on the outside of the building.

The arrangement of the bed-rooms and galleries is a modification of the single and the double-sided system—viz., of wards which have bed-rooms on one side only, and those which have bed-rooms on both sides. By the adoption of this plan, the asylum would not cover so large an area as it would if constructed with the single row of bed-rooms throughout, and it tends materially to keep down the expense of erection. The galleries are eleven feet in width, with a large window at the extreme end of each; these windows, together with open compartments for taking the meals, leave the asylum perfectly free from dark corners, and at the same time give a cheerful view of

* Motto—"Humanitas."

† Motto—"Practice with Theory."

the surrounding country. By this arrangement, there are no nooks or hiding-places, and it is favourable for inspection.

The whole of the building was proposed to be fire-proof, the stories being separated by brick arches, and the roofs, sashes, and shutters formed of cast or wrought-iron. The style adopted is Tudor, the materials red brick, with white-brick dressings, excepting the central portion, where stone would be used, and the estimated cost was 82,000*l*. For a three-story building the cost was put at 75,000*l*.

The authors of the third design (Messrs. Allom and Crosse,* premium 100*l*.) appear to have inclined to a three-story building. The plan, as in number 1, presents an extended straight line of building, with a wing at each end projecting towards the south, and then again extending towards the east on one side, and towards the west on the other. The wards are mostly double. The style is Elizabethan, the materials red brick, with white brick or stone dressings; and the estimated cost, if we understood rightly, also 75,000*l*.—a curious coincidence, considering that no amount was stipulated by the magistrates. The plan for affording accommodation in a two-story structure shows a square pile of building at each end of the main line, surrounding an open quadrangle.

There were several very clever designs amongst the rejected plans, but the majority of them were wholly unfit for the purpose, although set forth with much artistic ability at great cost. They showed a fearful waste of time and money.

